# A free boundary problem-in time-for the spread of Covid-19

**DOI:** 10.1007/s00285-023-01881-0

**Published:** 2023-02-15

**Authors:** Stephan Luckhaus, Angela Stevens

**Affiliations:** 1 Mathematical Institute, University of Leipzig, Augustusplatz 10, D-04109 Leipzig, Germany; 2 Institute for Analysis and Numerics, University of Münster, Einsteinstr. 62, D-48149 Münster, Germany

**Keywords:** Covid-19, Delay-differential equations, Kinetic equations, 45D05, 82D99, 92D30, 34K04

## Abstract

In this paper we deal with two aspects of the Covid epidemic. The first is a phase change during the epidemic. The empirical observation is that once a certain threshold of active infections is reached, the rate of infection is increasing significantly. This threshold depends, among others, also on the season. We model this phenomenon as a jump in the coefficient of the virus exposition, giving the force of infection. In a chemical mass action law this coefficient corresponds to the reaction rate. We get a free boundary problem in time, which exhibits deterministic ‘metastability’. In a population which is in a state of herd immunity, still, if the number of imported infections is large enough, an epidemic wave can start. The second aspect is the two scale nature of the infection network. On one hand side, there is always a finite number of reoccuring–deterministic–contacts, and on the other hand there is a large number of possible random contacts. We present a simple example, where the group size of deterministic contacts is two, and the graph of random contacts is complete.

## Introduction

In this paper we deal with the Covid epidemic and two aspects, which are not covered by “classical” Kermack-McKendrick models.

The first is a nonlinearity in the force of infection, which we model as a “ree boundary”. Let us explain this ansatz. The probability to get infected during a random encounter with one or several infectious individuals depends on the amount of virus inhaled. This dependence is usually thought of as a dose response curve of concave, convex shape. In order to get the expected per capita rate of infection–the force of infection–in principle, one has to evaluate the expectation of this function w.r.t. the probability distribution for the virus exposition at a random encounter, see e.g. (Luckhaus 2020), Ch. 5. We call *I* the expected virus exposition, which in turn is a linear integral operator in the distribution of time since infection, *a*, in the population. One may expect to keep properties like the convex, concave shape, and monotonicity obviously, and approximate the force of infection *f* with a piecewise linear function. In the present paper we assume that the convex part of *f* is never visited during the evolution, which is probably realistic, and consider just one jump in $f'(I)$. How this fits observations for Covid is discussed in the next section. One of the advantages of this ansatz is that even though one loses an invariant of the evolution, which one has for linear *f*, the corresponding quantity is piecewise constant in time. It jumps at the times $t_j$ where $f'(I)$ jumps, i.e. where *I* crosses a certain threshold. These points $t_j$ mark what we call a free boundary in time.

The phase change in the epidemic occurs at a certain threshold for the ratio of infections in the population, also called incidence. For a large metropolitan area during the flu season this threshold might be a rate of about $5-10\%$ infections per month for Covid in the active part of the population. We will comment on this number below.

The second aspect we deal with is heterogeneity of the infection process. Infection can take place in a group with a deterministic contact pattern or during random encounters as mentioned above. For these processes we introduced a fairly general hands on approach in Luckhaus and Stevens (2023) leading to a McKendrick type system. It is based on empirical laws $\alpha (a), \beta (a)$ for individual, respectively in group infectiousness, coupling the time *a* elapsed since infection to virus exposition within one’s own group or at a random encounter. Here we deal with the simplest example, a group of two indistinguishable individuals, one variant of the virus, and no re-infection. This model can be thought of as a proof of concept. But one can easily generalize it to re-infections, different virus variants, and so forth.

In such a case the possible sequences of in group infections are described in the normal form of game theory. This goes back to the theory that John von Neumann proposed in a talk of the Göttingen academy in 1926 (Neumann 1928), the same year, during which also A. G. McKendrick proposed the Kermack-McKendrick model (McKendrick 1926). In this case one has a tree whose nodes correspond to a particular sequence of infections that have occurred, and the edges correspond to a move by the player “chance”, who is infecting the next member of the group.

There is also an evolution taking place at the nodes. This corresponds to the independent evolution of the infectiousness of the individuals and the continuous losses by the moves of the player “chance”. So at each node one has a kinetic equation, as it was proposed by McKendrick (1914). More generally, this equation could be replaced by other semilinear PDEs, corresponding to different independent processes.

**Fig. 1:**
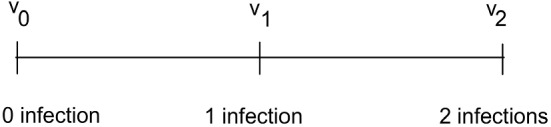
On the edge $(v_0, v_1)$ one of two susceptibles gets infected. On $(v_1,v_2)$ the other one

In this paper, we keep things as simple as possible. Let the group consist of two indistinguishable individuals, so the resulting tree is just a sequence of 3 nodes, see Fig. 1. And there is one phase change, a jump in the derivative of the force of infection for the random contacts as mentioned above. We call it a phase change primarily in analogy to chemical reactions, e.g. of combustion type, where the reaction rate, the coefficient of the mass action law, has an abrupt change (mediated by the heat created in this case). Since in each group there are two individuals, the state space of the groups–as McKendrick would have called it–is two-dimensional, i.e. the two times elapsed since the respective infections. And also the manifold of stationary states is two-dimensional.

In Fig. 1 on the edge $(v_0, v_1)$ one of the two susceptible individuals gets infected. On the edge $(v_1,v_2)$ the remaining susceptible individual gets infected.

The models introduced in Luckhaus and Stevens (2023) are not the first ones in epidemiology having a two level or group structure, see e.g. (Becker and Dietz 1995). And especially in the community working on stochastic epidemiological models, there is a lot of research on heterogeneous infection structures. Let us quote (Pardoux 2016) as a general reference, (Backhausz and Balázs 2022) for general contact graph structures, and (Forien and Pardoux 2022) for a household model of SIS type but otherwise similar to the process we discuss. The stochastic aspects are not subject of the present paper, even though stochastic epidemiology has a long history too (Feller 1939). In the stochastic literature the results are nowadays often described by SDEs. We start directly from what would be the hydrodynamic limit, i.e. a limit where the initial number of infections is proportional to *N*, the population size, and $N\rightarrow \infty$.

For us it is important to be able to use empirical laws for the evolution of infectiousness, which can be adapted to what was measured e.g. with PCR-tests in Zou et al. (2020).

That said, one of the results of the present paper is that for the model we discuss here, we could find a second invariant–this one for general *f*(*I*). Then the outcome of an epidemic starting at an unstable stationary point with a proportion of *s* uninfected groups and a proportion $r_1$ of groups with one immunized individual is determined by very few–namely 3–parameters of the infection process and the values of *s* at the “free boundary” points only, i.e. $s_1$ and $s_2$ in formula (6). This formula describes explicitly the relative influence on the outcome of the epidemics of the in group infection–$\beta$ in the formula, the exposition of random encounters, and the coefficient jump at the free boundary.

For an arbitrary initial datum we also have a formula which describes the outcome of the epidemic. The initial datum is the ‘age structure’ of the active infections, i.e. the distribution of infection times over a finite interval. We always assume that infected individuals stay infectious only for a limited amount of time. Using two invariants of the evolution, the outcome of the epidemic is determined by the ratio of groups with one, respectively both individuals removed, the values of the ratio *s* of susceptible groups with no infection at the free boundary points, and four integral expressions in the distribution of active infections. This finite data set does not determine the evolution, it determines only at which point in the two dimensional manifold of stable stationary states the epidemic finally ends.

What we cannot discuss in this deterministic context is how an epidemic can be started by a finite number of infected individuals. But it is clear which result to expect. If the population is in a stable state, a state of “herd immunity”, then with probability one, an infinite number of infections is never reached. If one starts in an unstable state on the other hand, there is a positive $\rho$ and a positive $p_0$, such that the probability to reach $\rho N$ infections at some time $t_N$ is larger than $p_0$, independent of the population size *N*. Here $t_N$ though grows like $\log (N)$.

McKendrick models in epidemiology are now often called age-structured population models and treated in the framework of delay-differential-equations, see (Hale 1977; Thieme 2003; Rass and Radcliffe 2003; Diekmann et al. 2012). We follow this approach in Sect. 4. The original existence proof for their system was given by Kermack and McKendrick, making use of the Paley-Wiener theorem, and we take the opportunity to quote this result here (Kermack and McKendrick 1936). In Sect. 2 we discuss data. In Sect. 3 we present a modification of the Kermack–McKendrick model.

## Data supporting the need for modifying the Kermack–McKendrick equations

As mentioned above, a phase change can be observed in Covid epidemic waves. The first indicator that $f'(I)$ cannot be assumed to be constant, as it would be the case in a standard contact process, is the observed metastability of the process. Take for instance Switzerland in spring 2020. Whereas in Geneva and Ticino $10\%$ and more of the population was infected, in Zurich the level of infections was much lower–probably by a factor of 10. Data are published on the website of the Bundesamt für Gesundheit (see link in references). This is what we try to explain by a “phase-change” in the infection process. And this has a physiological explanation. It is not just a change in *f*(*I*), it is also seen e.g. in a change of mortality, or as it is technically called, the infection fatality risk (IFR).

When a susceptible individual inhales the virus, the probability of getting infected depends crucially and nonlinearly on the virus load that reaches the pharynx. The probability to actually get ill depends crucially on the virus load deposited in the lungs. This is the reason why during experiments with macaques the virus is directly introduced into the monkeys’ windpipes (Chandrashekar et al. 2020), otherwise they would not get symptomatically infected. High infection rates and significant symptoms have also been observed in party-like super spreading events. A striking example happened on 3rd of September 2021, when ca. 400 individuals, about 20–30 years old and with a shortly before acquired fully vaccinated status met in a club in Münster, Germany (Westfälische Nachrichten 2021). By September 9th, 26 participants had seen doctors and had PCR-confirmed infections. That means of course that their experienced symptoms cannot have been mild, since people were not expecting to fall ill, but believed to be protected by the vaccination. In the end 85 persons tested positive.

So the force of infection and the ratio of people getting severely ill depend both nonlinearly on the number of infectious in the population.

So what are the numbers that we have for the IFR and the incidence. One of the best researched early epidemic waves is the april 2020 one in Geneva. The SeroCovPop study (Stringhini et al. 2020) found ca. $10 \%$ seropositivity in the active population. With todays consensus estimate of $80\%$ seroconversion,^1^ according to what the head of the AGES, the austrian public health service, stated in an interview in June 2021 (Allerberger 2021), one arrives at ca. $12\%$ infections within the population between mid march and mid may 2020. (For an estimate on the seroconversion rate based on the ratio of asymptomatic cases among the seroconverted, we refer to Luckhaus (2020)). This is one of the lowest infection ratios per month, for which one definitely could observe an epidemic wave. The corresponding overall mortality–the infection fatality risk (IFR)–was estimated as 0.6–0.7% (Perez-Saez et al. 2020). If you take into account the above lower seroconversion rate, and strip out the cases in nursing homes and ambulant care (together $\approx 60\%$ of all deaths), you would still have a probability of 0.2–0.3% to die during a Covid infection, which is also the estimate for Ischgl (Knabel et al. 2021).

Let us compare this to the Pfizer study (Thomas et al. 2021):

The study was planned as a 6 month triple blind study with ca. 40.000 participants. It is debatable whether the claimed vaccine efficacy is reliable (Thacker 2021). For one thing no attempt was made to estimate the number of asymptomatic infections (Rubin and Longo 2020). Within the group of the recipients of the placebo, two deaths with positive PCR-test were counted among the ca. 1000 self reported symptomatic cases. To get the IFR, one has to divide roughly by 10 (see e.g. Ischgl (Knabel et al. 2021) or the CRO study discussed below) in order to account for the asymptomatic and pauci symptomatic infections. So mortality in the placebo group of the Pfizer study was about one tenth of the comparable mortality in Geneva or Ischgl. And in the Pfizer study $40\%$ of the participants were over 55-years old. That would mean that the IFR of this age group having obtained the placebo would be $0.05\%$, whereas in Geneva the IFR for the age group 50–64 was calculated to be $0.14\%$, and adjusting for lower seroconversion than was assumed in Perez-Saez et al. (2020), it still would be $0.1 \%$, see (Luckhaus 2020). This is a significant difference in the IFR pointing to a qualitative change in the infection.

Of course it is not only the ratio of active infections which plays a role. Also the season does play a role. The bulk of the observations in the clinical trial NCT04368728, the Pfizer trial, refers to the beginning of august to mid of december 2020. So there is only a small overlap with what is generally the flu season, i.e. the winter season e.g. in middle Europe, during which the IFR is higher than during summer.

The other study we want to quote had as its object the infection risk in public transport in a region close Frankfurt airport in the calendar weeks 7–12 of 2021. The report on the study was prepared by CRO, the Charité Research Organisation and was published on the website of the Ministry of Traffic of the german federal state of Baden Württemberg (Charité Research Organisaton 2021). 661 individuals, who had tested negative in the Euro Immune IgG-test as well as the PCR-test, were followed up for 4 weeks with questionnaires, and they were tested again after 5 weeks. Three PCR-positives were found during the above 4 weeks, only one more person was found by the final PCR-test, but 23 more were found by the final IgG-test. That gives again ca. $10\%$ of the infectious found by self-reports. But it also shows that PCR-visibility of the 23 seropositives was extremely short, making it almost certain that these individuals, though they had been seronegative at the start of the study, had already been infected with the Wuhan variant of Covid-19 and now were re-infected with the UK-variant of the virus.

This requires an explanation. It shows that in spite of the lockdown in force in Germany since mid of november 2020, the majority of the active population in a large metropolitan area had been–asymptomatically–infected.

And this is what we try to explain with an increase of *in group infection*, offsetting at least partially the reduction of random contacts in public transport, department stores, pubs, cinemas, concerts etc. In Sects. 4 and 5 we discuss how this effect looks like for our example of a two person group, and how it becomes stronger for larger groups.

Coming back to IFRs: To have a more precise information about IFRs would be very helpful for a better epidemiological understanding of the Covid-19 epidemics. After all, the number of Covid deaths is a “hard” statistical datum. And most countries use the WHO-definition, where Covid deaths are all deaths with a positive PCR-test up to 28 (in Sweden 30) days before death. And the IFR is after all the ratio of these Covid deaths among all infections.

There are exceptions, e.g. Singapore, where only people with Covid as a primary cause of death are counted, or Germany, where the Robert Koch Institute (RKI) validates the death certificates of the under 20-years old, and eliminates eventually cases, where Covid was not a cause of death, but a coincidental infection (“Begleitinfektion”).

The IFR is unfortunately not constant. It depends on the season, the virus variant of Covid, and primarily on age. Our impression is that the IFR of the age group between 50 and 60 years old is probably the most useful, in order to obtain the number of Covid infected during an epidemic wave. This is the case, because the over 70 years old include the cases in nursing homes, and the percentage of elderly living in nursing homes varies quite a lot. For the under 50 years old, Covid deaths are extremely rare, two in the case of Geneva, and reliable estimates for Covid incidence based on IFR are possible only for large populations.

Further, there is a strong indication, that the IFR has decreased significantly with the $\alpha$-variant of the virus, and now with omicron in the older age groups. For the younger age groups the contrary is the case. Just to give the example for the $10-19$-years old in Germany (data from RKI): During the first two epidemic waves, caused by the Wuhan variant, there were just 2–validated and male–Covid deaths (up to february 16, 2021). Now in 2022 with omicron in a largely vaccinated teenage population between february 16, 2022 and august 31, 2022 we have 4 new male and 12 new female Covid deaths in the statistics. The RKI does no longer give the information how many of these cases are validated, but the reported numbers were unchanged for 3 weeks.

Judging from the RKI data, in the age group $50-60$ the IFR seems to be more stable. Up to february 16, 2021, after two epidemic waves, one in winter, 1667 deaths appeared in the statistics. Between february 16, 2022 and august 31, 2022, 841 new deaths appeared in the statistics. So the IFR now is most probably at least half the one for the Wuhan type epidemic. Note, that the decimation of the vulnerable part of the population is completely irrelevant for the spreading of the virus, but extremely relevant for the change in the IFR.

## The modified Kermack–McKendrick model

As explained in the introduction, we start from a population of groups, each consisting of two indistinguishable individuals. After the first infection these become groups $i_1$ with one infected, characterized by one parameter *a*, namely the time elapsed since infection.

The second infection can be either an in group infection occurring with a rate $\beta (a)$, which depends on *a* only. Or it can be an infection incurred in a random meeting outside the group depending on the expected virus load at this meeting, which again depends on the proportion of infectious in the population. The groups $i_2$ with two infected are characterized by the time $a_1$ elapsed since the second infection and the time $a_2$ elapsed since the first infection. The expected per capita infection rate for a random meeting will be denoted by *f*(*I*), where *f* is monotone and *I* is a weighted mean of the density of infected groups. The contribution of an individual who has been infected at the time $t-a$ is denoted by $\alpha (a)$. Then we get the $s-i$-system written below, with susceptibles *s* and infected $i_1, i_2$. To be precise $s,i_1,i_2$ are percentages of groups in the total population of groups.

$$\begin{aligned} s(\cdot ) + \int _0^\infty i_1(a,\cdot ) \; da + \int _0^\infty \int _{a_1}^\infty i_2(a_1,a_2,\cdot ) \; da_2 \; da_1 \equiv 1 \;. \end{aligned}$$

In the following, derivatives have to be understood in the weak sense.

1 $$\begin{aligned}{} & {} \partial _t s(t) = -2 s(t) f(I(t)) \quad \text{ with } \text{ the } 2 \text{ here, } \text{ since } \text{ individual } 1 \text{ or } 2 \text{ can } \text{ be } \text{ infected } \nonumber \\{} & {} \partial _t i_1(a,t) + \partial _a i_1(a,t) = - i_1(a,t) \left[ \beta (a) + f(I(t)) \right] \;, \text{ in } 0< a< \infty \nonumber \\{} & {} i_1(0,t) = - \partial _t s(t) \nonumber \\{} & {} \partial _t i_2(a_1,a_2,t) + \partial _{a_1} i_2 (a_1,a_2,t) + \partial _{a_2} i_2(a_1,a_2,t) = 0 \; \text{ in } 0<a_1<a_2< \infty \nonumber \\{} & {} i_2(0,a,t) = - \partial _t i_1(a,t) - \partial _a i_1(a,t) \nonumber \\{} & {} I(t) = \int _0^\infty \alpha (a) i_1(a,t) \; da + \int _0^\infty \int _{a_1}^\infty \big ( \alpha (a_1) + \alpha (a_2) \big ) i_2 (a_1, a_2, t) \; da_2\; da_1 \;.\nonumber \\ \end{aligned}$$

Also initial conditions at $t=0$ for $s, i_1(a,.)$ and $i_2(a_1,a_2,.)$ can be specified, if one wants to solve the initial boundary value problem. In the following we concentrate on the heteroclinic orbit, connecting two stationary points. It is useful to calculate the time derivative of a general weighted mean $\partial _t \Gamma$ of the population density of the infected groups in the $s-i$-model via integration by parts. This allows to compute invariants and to transform the kinetic equations into delay differential equations with distributed delay. In order to do so, define

$$\begin{aligned} \Gamma (t) = \int _0^\infty \gamma _1(a) i_1(a,t) \; da + \int _0^\infty \int _{a_1}^\infty \gamma _2(a_1, a_2) i_2(a_1, a_2,t) \; da_2 \; da_1 \;. \end{aligned}$$

with weights $\gamma _1$ and $\gamma _2$. Then

2 $$\begin{aligned} \partial _t \Gamma (t)= & {} \int _0^\infty \gamma _1 (\partial _t + \partial _a) i_1 \; da +\int _0^\infty (\partial _a \gamma _1) i_1 \; da + \gamma _1 (0) i_1(0,t) \nonumber \\{} & {} + \int _0^\infty \int _{a_1}^\infty \Big ( (\partial _{a_1} + \partial _{a_2}) \gamma _2 \Big ) i_2 \; da_2 da_1 + \int _0^\infty \gamma _2(0,a) i_2(0,a,t) \; da \nonumber \\= & {} \int _0^\infty \Big (- \gamma _1(a) + \gamma _2(0,a)\Big ) \Big ( \beta (a) + f(I(t))\Big ) i_1(a,t) \; da + \gamma _1(0) 2 s(t) f(I(t)) \nonumber \\{} & {} + \int _0^\infty (\partial _a \gamma _1) i_1 \; da + \int _0^\infty \int _{a_1}^\infty \Big ( (\partial _{a_1} + \partial _{a_2} ) \gamma _2 \Big ) i_2 \; da_2 \; da_1 \;. \end{aligned}$$

Note, that this is a formal calculation corresponding to the use of test functions $\gamma _1(a) \phi (t)$ and $\gamma _2(a_1,a_2) \psi (t)$. If *f* is the identity, this shows that

$$\begin{aligned} \log s(t) + 2 \left[ \int _0^\infty \gamma (a) i_1(a,t) \; da + \int _0^\infty \int _{a_1}^\infty \big ( \gamma (a_1) + \gamma (a_2) \big ) i_2 (a_1, a_2,t) \; da_2 \; da_1 \right] \end{aligned}$$

with $\gamma (a) = \int _0^a \alpha (\sigma ) \; d\sigma$, is an invariant of the evolution.

Invariants can be seen as restrictions on possible epidemic curves, for instance heteroclinic orbits connecting an unstable stationary point to a stable one. Thus they allow to link global data of an epidemic with not so easily observable local rate dependencies. In the classical Kermack-McKendrick model with age structure there is one invariant and a one-parameter family of stationary points. This allows to write a scalar equation for the final size of the epidemic. Similarly, for a model with *n* subpopulations one has *n* invariants, a $n \times n$ system of equations which determines the outcome of the epidemic, see (Rass and Radcliffe 2003). Also in our situation we were able to find a second invariant of the evolution, and consequently for linear *f* a formula determining the outcome of an epidemic wave. This will be discussed in the next section.

Formula (2) also allows to get an equation for $\partial _t I(t)$. There is always a delay between getting infected and becoming infectious. For Covid this is ca. two days, and it takes one day more for the onset of symptoms (Luckhaus 2020). So we have $\alpha (0) = 0$ and

$$\begin{aligned} \partial _t I(t) = \int _0^\infty \alpha '(a) i_1(a,t) \; da + \int _0^\infty \int _{a_1}^\infty \Big (\alpha '(a_1) + \alpha '(a_2)\Big ) i_2(a_1, a_2,t) \; da_2 \; da_1 , \end{aligned}$$

where we write $\alpha '(a)$ for the derivative of $\alpha$ w.r.t. *a*.

## An sir-formulation as a delay differential equation with distributed bounded delay

In the $s-i$-formulation above the only stationary point is $s=0$, because there is a spurious evolution for $i_1$ and $i_2$ without influence on *I*, the proxy for the virus load.

In order to introduce the notion of removed groups, we choose a cutoff function $\eta :\mathbb {R}_0^+ \rightarrow [0,1]$ with compact support, $\eta '(a) \le 0$ for all $a \in \mathbb {R}_0^+$, and such that $(1-\eta ) \alpha = (1- \eta ) \beta \equiv 0$. We define the following quantities

$$\begin{aligned} r_1(t)= & {} \int _0^\infty (1- \eta (a) ) \; i_1(a,t) \; da \\ r_2(t)= & {} \int _0^\infty \int _0^\infty (1- \eta (a)) \; i_2 (a,\sigma ,t) \; d\sigma \; da \\ i_3(a,t)= & {} \int _a^\infty (1- \eta (\sigma - a)) \; i_2(a,\sigma ,t) \; d\sigma . \end{aligned}$$

Here $r_1$ is the fraction of those groups with one removed individual and one susceptible, and $r_2$ the fraction of the groups with two removed individuals. Note that $r_1(t), r_2(t)$ and $i_3(a,t)$ depend on the choice of $\eta$ but the limit for $t\rightarrow \infty$, $r_1(\infty ), r_2(\infty )$ does not. Then, by formal calculations, which again can be made rigorous by inserting test functions in the kinetic equations, we get

$$\begin{aligned} \partial _t r_1(t)= & {} - \int _0^\infty \eta '(a) i_1(a,t) \; da - f(I(t)) r_1(t) \\ (\partial _t + \partial _a) i_3(a,t)= & {} 0 \quad , \quad i_3(0,t) = f(I(t)) r_1(t) \\ \partial _t r_2(t)= & {} - \int _0^\infty \int _0^\infty \eta '(a) i_2(a,\sigma , t) \; d\sigma \; da \\= & {} - \int _0^\infty \int _a^\infty \eta '(a) \eta (\sigma -a ) i_2(a, \sigma , t ) \; d\sigma \; da - \int _0^\infty \eta '(a) i_3(a,t) \; da \\ I(t)= & {} \int _0^\infty i_1(a,t) \alpha (a) \; da + \int _0^\infty \int _a^\infty i_2(a,\sigma ,t) \alpha (\sigma ) \; d\sigma \; da \\{} & {} + \int _0^\infty \int _a^\infty i_2(a, \sigma , t) \alpha (a) \eta (\sigma - a) \; d\sigma \; da + \int _0^\infty i_3(a,t) \alpha (a) \; da . \end{aligned}$$

Using the identity

$$\begin{aligned} \left( \partial _t + \partial _a \right) \log \Big ( i_1(a,t) \frac{1}{\sqrt{s(t)}} \exp (b(a)) \Big ) = 0 \quad \text{ where } \quad b(a) = \int _0^a \beta (\sigma ) \; d\sigma \;, \end{aligned}$$

one arrives at

3 $$\begin{aligned} i_1(a,t)= & {} \frac{i_1(0,t-a)}{\sqrt{s(t-a)}} \sqrt{s(t)} \exp (-b(a)) \\= & {} 2 \sqrt{s(t)} \sqrt{s(t-a)} f(I(t-a)) \exp (-b(a)) \nonumber \\ i_2(a_1,a_2,t)= & {} i_2(0,a_2 - a_1, t-a_1) = i_1(a_2 - a_1, t-a_1) \Big [ \beta (a_2 - a_1) + f(I(t-a_1)) \Big ] \nonumber \\ i_3(a,t)= & {} i_3(0,t-a) = r_1(t-a) f(I(t-a)) . \nonumber \end{aligned}$$

First we get a (second) invariant:

$$\begin{aligned}{} & {} \partial _t \left( \frac{r_1}{\sqrt{s}} - 2 \exp (- b(\infty )) \int _0^\infty \sqrt{s(t-a)} \eta '(a) \; da \right) \\{} & {} \quad \quad \quad = \frac{\partial _t r_1}{\sqrt{s}} - \frac{1}{2} \frac{r_1}{\sqrt{s}} \frac{\partial _t s}{s} - \exp (- b(\infty )) \int _0^\infty \frac{\partial _t s(t-a)}{\sqrt{s(t-a)}} \eta '(a) \; da = 0 \end{aligned}$$

since the third term of the sum on the right hand side equals

$$\begin{aligned} \int _0^\infty \sqrt{s(t-a)} 2 f (I(t-a)) \exp (-b(a)) \eta '(a) \; da = \frac{1}{\sqrt{s(t)}} \int _0^\infty i_1(a,t) \eta '(a) \; da . \end{aligned}$$

And finally we get the delay differential system

4 $$\begin{aligned} \partial _t s(t)= & {} - 2 s(t) f(I(t)) \nonumber \\ \partial _t r_1(t)= & {} - r_1(t) f(I(t)) + 2 \sqrt{s(t)} \exp (-b(\infty )) \int _0^\infty \sqrt{s(t-a)} f(I(t-a)) \eta '(a) \; da \nonumber \\ \partial _t I(t)= & {} \int _0^\infty \alpha '(a) i_1(a,t) \; da + \int _0^\infty \alpha '(a) i_3(a,t) \; da \\{} & {} + \int _0^\infty \int _0^\infty \Big ( \alpha '(a + \sigma ) + \alpha ' (a) \eta (\sigma ) \Big ) \beta (\sigma ) i_1(\sigma , t-a) \; d\sigma \; da \nonumber \\{} & {} + \int _0^\infty \int _0^\infty \Big ( \alpha '(a + \sigma ) + \alpha '(a) \eta (\sigma ) \Big ) f(I(t-a)) i_1(\sigma , t-a) \; d\sigma \; da \nonumber \end{aligned}$$

Inserting the identities for $i_1$ and $i_3$ leads to a delay differential system with bounded distributed delay in the variables $s,r_1, I$. All points with $\int _0^\infty \int _0^\infty \eta (a) \eta (\sigma )I(t-\sigma -a) \; da \; d\sigma = 0$ are stationary points, and the theory of functional differential equations or ODEs in Banach spaces (Hale 1977) applies. In order to linearize the system it is convenient to go back to the kinetic formulation (1). If one linearizes around $r_1(0), s(0), r_2(0)=1-r_1(0) - s(0)$, then the linearization $\hat{s}, \hat{r}_1, \hat{i}_1, \hat{i}_2, \hat{i}_3$ satisfies

$$\begin{aligned} \partial _t \hat{s}(t)= & {} -2 s(0) f'(0) \hat{I}(t) \\ (\partial _t + \partial _a) \hat{i}_1(a,t)= & {} - \beta (a) \hat{i}_1(a,t), \quad \hat{i}_1(0,t) = 2 s(0) f'(0) \hat{I}(t) \\ (\partial _t + \partial _{a_1} + \partial _{a_2} ) \hat{i}_2(a_1, a_2, t)= & {} 0, \quad \hat{i}_2(0,a.t)=\beta (a) \hat{i}_1(a,t) \\ (\partial _t + \partial _a) \hat{i}_3(t)= & {} 0, \quad \hat{i}_3(0,t) = r_1(0) f'(0) \hat{I}(t) \\ \partial _t \hat{r}_1(t)= & {} - r_1(0) f'(0) \hat{I}(t) \\{} & {} + 2 s(0) \exp (-b(\infty )) \int _0^\infty f'(0) \hat{I}(t-a) \eta '(a) \; da \\ \hat{I}(t)= & {} \int _0^\infty \alpha (a) \hat{i}_1(a,t) \; da + \int _0^\infty \alpha (a) \hat{i}_3(a,t) \; da \\{} & {} + \int _0^\infty \int _{a_1}^\infty [ \alpha (a_1) \eta (a_2 -a_1) + \alpha (a_2)] \hat{i}_2(a_1, a_2, t) \; da_2 \; da_1 \end{aligned}$$

Since there is no feedback by $\hat{r}_1$ to the other equations, we omit $\partial _t \hat{r}_1$ in the following. The linearized system actually decouples and is equivalent to the linearization of a scalar McKendrick equation

$$\begin{aligned}{} & {} (\partial _t + \partial _a) \tilde{i}_1 (a,t) = 0,\quad \text{ with } \quad \tilde{i}_1 (a,t) = \exp (b(a)) \hat{i}_1(a,t) \\{} & {} (\partial _t + \partial _a) \hat{i}_3(a,t) = 0 , \quad (\partial _t + \partial _{a_1} + \partial _{a_2}) \hat{i}_2(a_1,a_2,t) = 0 \\{} & {} \tilde{i}_1(0,t) = 2 s(0) f'(0) \hat{I}(t), \quad \hat{i}_3(0,t) = r_1(0) f'(0) \hat{I}(t), \quad \hat{i}_2(0,a,t) = \beta (a) \hat{i}_1(a,t) \\{} & {} \hat{I}(t) = \int _0^\infty \alpha (a) \left[ \hat{i}_1(a,t) + \hat{i}_3(a,t) \right] \; da \\{} & {} \qquad \qquad + \int _0^\infty \int _0^\infty \left( \alpha (a_1) + \alpha (a_1 + \sigma ) \right) \hat{i}_2(a_1, a_1 + \sigma , t ) \; da \; d\sigma . \end{aligned}$$

Respectively

$$\begin{aligned} \hat{I}(t)= & {} \int _0^\infty \alpha (a) \left[ \exp (-b(a)) \tilde{i}_1(a,t) + \hat{i}_3(a,t) \right] \\{} & {} + \int _0^\infty \int _0^\infty \left[ \alpha (a) + \alpha (a + \sigma ) \right] \beta (\sigma ) \exp (-b(\sigma )) \tilde{i}_1 (\sigma , t-a) \; da \; d\sigma \;. \end{aligned}$$

The characteristic equation for the leading (real) eigenvalue becomes

$$\begin{aligned}{} & {} \partial _a \hat{i}_1 = (-\lambda -\beta (a)) \hat{i}_1, \quad \partial _a \hat{i}_3 = - \lambda \hat{i}_3 , \quad (\partial _{a_1} + \partial _{a_2}) \hat{i}_2 = - \lambda \hat{i}_2 \\{} & {} \hat{i}_1 (0) = 2 s(0) f'(0) \hat{I} , \quad \hat{i}_3(0) = r_1(0) f'(0) \hat{I} , \quad \hat{i}_2(0,a) = \beta (a) \hat{i}_1 (a) \\{} & {} 2 s(0) f'(0) \int _0^\infty \alpha (a) \exp (-\lambda - \beta (a)) \; da + r_1(0) f'(0) \int _0^\infty \alpha (a) \exp (- \lambda a) \; da \\{} & {} \qquad + 2 s(0) f'(0) \int _0^\infty \int _0^\infty \exp (- \lambda a) \beta (\sigma ) \exp ( -\lambda \sigma - b(\sigma )) \big (\alpha (a) + \alpha (a +\sigma ) \big ) \; da \; d\sigma = 1 \end{aligned}$$

We have used, that $\eta (\sigma ) \beta (\sigma ) = \beta (\sigma )$. The boundary of the stability region is given by $\lambda = 0$, which simplifies to

$$\begin{aligned} r_1(0) \gamma (\infty ) f'(0) + 2 s(0) f'(0) \gamma (\infty ) \left( 1- \exp (-b(\infty ))\right) = 1 . \end{aligned}$$

In the special case $f(I)= I + \delta \big [ I - I_0 \big ]_+$ with $\delta$ representing the jump in the infection rate, and $I_0$ being a constant, the quantity which was an invariant for linear *f*, now jumps at the points where *I*(*t*) crosses $I_0$. More precisely with $\Gamma$ defined by

$$\begin{aligned} \Gamma (t)= & {} \hat{\Gamma }(t) + 2 \gamma (\infty ) r_2(t) + \gamma (\infty ) r_1(t) \text{ where }\\ \hat{\Gamma }(t)= & {} \int _0^\infty \gamma (a) \eta (a) i_1(a,t) \; da \\{} & {} + \int _0^\infty \int _0^\infty (\gamma (a) + \gamma (a+\sigma )) \eta (a) \eta (\sigma ) i_2(a,a+\sigma ,t) \; da d\sigma \\{} & {} + \int _0^\infty \big (\gamma (a) + \gamma (\infty )\big ) \eta (a) i_3(a,t) \; da \end{aligned}$$

and $\gamma (a) = \int _0^a \alpha (\sigma ) \; d\sigma$ we have

$$\begin{aligned} \partial _t \Big ( \Gamma + \frac{1}{2} \log s \Big )= & {} 0 \quad \text{ for } \quad I \le I_0 \\ \partial _t \Big ( \Gamma + \frac{1}{2}(1 + \delta ) \log s \Big )= & {} 0 \quad \text{ for } \quad I > I_0 \;. \end{aligned}$$

The similarity with free boundary problems becomes evident if one formally rewrites the above as

5 $$\begin{aligned} \partial _t \left( \Gamma + \frac{1}{2} \log s + \frac{\delta }{2} H(I- I_0) \log s\right) = \frac{\delta }{2} \partial _t \big (H (I - I_0)\big ) \log s , \end{aligned}$$

where *H* is the Heaviside function and the equation has to be understood in the distributional sense. Consequently, if an epidemic wave starts e.g. at a stationary point with values $s_0, r_{1,0}$, then hits $y= I_0$ at $s_1$, and intersects $y=I_0$ at $s_2$ again, finally stopping at $s_\infty , r_{1,\infty }$, we get

6 $$\begin{aligned} \gamma (\infty ) \big ( r_{1,\infty } - r_{1,0} \big ) + 2\gamma (\infty ) \big ( r_{2,\infty } - r_{2,0} \big ) = \frac{1}{2} \log \left( \frac{s_0}{s_\infty } \right) + \frac{1}{2} \delta \log \left( \frac{s_1}{s_2} \right) , \end{aligned}$$

where $s_1$ and $s_2$ are the values of *s* at the "free boundary". Using the second invariant, i.e.

$$\begin{aligned} \frac{r_1(0)}{\sqrt{s(0)}} - 2 \exp (-b(\infty )) \sqrt{s(0)} = \frac{r_1(\infty )}{\sqrt{s(\infty )}} - 2 \exp (-b(\infty )) \sqrt{s(\infty )} \end{aligned}$$

respectively

$$\begin{aligned} r_1(\infty )= r_1(0) \sqrt{\frac{s(\infty )}{s(0)}} - 2 \sqrt{s(0)} \sqrt{s(\infty )} \exp (-b(\infty )) + 2 s(\infty ) \exp (-b(\infty )) , \end{aligned}$$

(6) then becomes $\phi \left( \sqrt{s(\infty )} / \sqrt{ s(0)} \right) = 0$ with

7 $$\begin{aligned} \phi (y)= & {} \frac{1}{2} \delta \log (s_2/s_1) + \log y + \gamma (\infty ) (r_1(0) + 2 s(0)) \nonumber \\{} & {} - 2\gamma (\infty ) s(0) [ 1 + \exp (-b(\infty ))] y^2 + \gamma (\infty ) [ 2 s(0) \exp (-b(\infty )) - r_1(0) ] y.\nonumber \\ \end{aligned}$$

And $\phi$ has exactly one stable zero, i.e. one zero with $\phi '$ being positive. This is the value of $\sqrt{s(\infty )} / \sqrt{s(0)}$ at the end of the epidemic.

In case $\delta =0$, $\beta =\infty$, $r_{1,0}= 0$, and $s_0=1$, one can directly compare the two-scale model with the standard Kermack-McKendrick model, see (Luckhaus 2020), with the same $\alpha$. One gets from the invariant

$$\begin{aligned} 4 \gamma (\infty ) (1- s_\infty ) + \log s_\infty = 0 \end{aligned}$$

for the two-scale model and

$$\begin{aligned} \gamma (\infty ) (1-s_\infty ) + \log s_\infty =0 \end{aligned}$$

for the classical model. That gives a crudely simplified and basic first idea of the effect of in group infections.

The invariants are useful not only to determine the heteroclinic orbits. They also link the outcome of an epidemic wave, starting from given initial data to these data. For this it is useful to rewrite the integral expression $\Gamma$ as

$$\begin{aligned} \Gamma (t)= & {} \Gamma _A(t) + 2 \gamma (\infty ) (r_1(t) + 2 r_2(t)) \quad \text{ where }\\ \Gamma _A(t):= & {} 2 \int _0^\infty \gamma (a) \eta (a) ( i_1(a,t) + i_3(a,t)) \; da \\{} & {} + \int _0^\infty \int _{a_1}^\infty (\gamma (a_1) + \gamma (a_2)) \eta (a_1 -a_2) \eta (a_1) i_2(a_1,a_2,t) \; da_2 \; da_1 \end{aligned}$$

separating the active infections from the removed.

$$\begin{aligned} 1-r_1(t) -r_2(t) - s(t) =: A(t)= & {} \int _0^\infty \eta (a) (i_1(a,t) + i_3(a,t)) \; da \\{} & {} + \int _0^\infty \int _{a_1}^\infty \eta (a_2-a_1) \eta (a_1) i_2(a_1,a_2,t) \; da_2 \; da_1 \end{aligned}$$

is a measure of the active infections. If one starts with $I(0) > I_0$ and supposes that $I(t) > I_0$, for $t < t_1$, $I(t) < I_0$ for $t < t_1$ then

$$\begin{aligned}{} & {} \Gamma _A(0) - 4 \gamma (\infty ) A(0) - 2 \gamma (\infty ) r_1(0) + 2 \gamma (\infty ) (1-s(0)) + \frac{1+\delta }{2} \log s(0) \\{} & {} \quad = \Gamma _A(t_1) - 4 \gamma (\infty ) A(t_1) - 2\gamma (\infty ) r_1(t_1) + 2 \gamma (\infty ) (1-s(t_1)) + \frac{1+\delta }{2} \log s(t_1) \\{} & {} \quad = \frac{\delta }{2} \log s(t_1) - 2 \gamma (\infty ) r_1(\infty ) + 2 \gamma (\infty ) (1- s(\infty )) + \frac{1}{2} \log s(\infty ) \end{aligned}$$

and

$$\begin{aligned} r_1(\infty ) = r_1(0) \sqrt{\frac{s(\infty )}{s(0)}} - 2 \exp (-b(\infty )) \left( s(\infty ) + \sqrt{s(\infty ) s(0)} \int _0^\infty \sqrt{\frac{s(-a)}{s(0)}} \eta '(a) \; da \right) \;. \end{aligned}$$

Or

8 $$\begin{aligned}{} & {} 2 \gamma (\infty ) (s(0) - s(\infty )) + \frac{1 + \delta }{2} \left( \log \frac{s(\infty )}{s(0)} \right) - \frac{\delta }{2}\left( \log \frac{s(t_1)}{s(\infty )} \right) \nonumber \\{} & {} \quad = \Gamma _A(0) - 4 \gamma (\infty ) A(0) - 2 \gamma (\infty ) r_1(0) \frac{s(0) - s(\infty )}{\sqrt{s(0) s(\infty )} + s(0)} \nonumber \\{} & {} \qquad +\, 4 \gamma (\infty ) \exp (-b(\infty )) \frac{s(0) - s(\infty ) }{\sqrt{s(0)} + \sqrt{s(\infty )}} \sqrt{s(\infty )}\nonumber \\{} & {} \qquad +\, 4 \gamma (\infty ) \exp (-b(\infty )) \int _0^\infty \left( \sqrt{s(\infty ) s(0)} - \sqrt{s(\infty )s(-a)} \right) \eta '(a) \; da \end{aligned}$$

This is no simple algebraic relation any more, since it contains the integral expressions $\Gamma _A, A, I$ and $\int _0^\infty \left( 1 - \sqrt{\frac{s(-a)}{s(0)}} \right) \eta '(a) \; da$, depending on the active infections. But these are just a few percent under realistic conditions.

## Discussion and generalization

The example of a two scale infection process which we have treated in detail above is minimal w.r.t. the group size. And the overall effect on the final size of the eventually removed individuals is limited by this. The parameters $\gamma (\infty ), b(\infty )$, and $\delta$ in formulas (6) and (7) have simple interpretations:

- $\gamma (\infty )$ is the total (aerosol) infectivity of an individual through random contacts,
- $b(\infty )$ is the total in group infectivity, and
- $\delta$ is the magnitude of the ’infectivity boost’ at the threshold incidence.

**Fig. 2:**
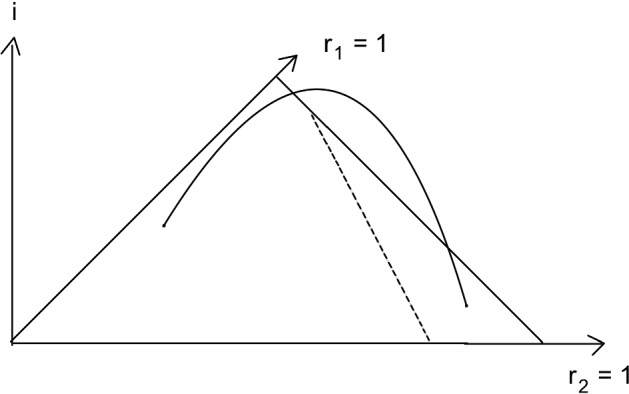
Example: heteroclinic orbit starting at unstable stationary point. $r_1 = 1$ is unstable. Broken line is boundary of stability region

If the groups are larger, the overall effect of the in group infection is increasing too, up to $N^2 \gamma (A)$, where *N* is the group size.

Fig. 2 shows an example of a heteroclinic orbit starting at an unstable stationary point, in a situation where $r_1 = 1$ is unstable. The variable *i* is a proxy for the variables $i_1(a) \eta (a)$, $i_3(a) \eta (a)$, $i_2(a_1,a_2) \eta (a_1) \eta (a_2 - a_1)$. So it lives in the Banach space $Y=\left( L^\infty \cap L^1\right) ^2(\mathbb {R}_0^+) \times \left( L^\infty \cap L^1 \right) ({\mathbb {R}_0^+}^2)$. The broken line is the boundary of the stability region.

Formula (7) gives an ‘algebraic’ relation between starting and end point of the heteroclinics. Of course this is at the cost of having to know $s_1$ and $s_2$, the values of *s* where the free boundary occurs. But our model also deals with the case that there is no heteroclinic orbit at all. Still starting with a certain amount of imported infectious individuals, an epidemic wave can be triggered. That is, what formula (8) describes, and what we think was the reason for the epidemic waves in in spring 2020 in the swiss cantons of Geneva and Ticino, where the ‘import’ came from France and Lombardia respectively .

Let us also comment on the use of these explicit formulas (except for the knowledge of *s* at the free boundary points). Abstractly speaking there is always a function $\Psi : \Omega \subset Y \times [0,1]^2 \rightarrow [0,1]^2$ foliating $\Omega$ by the outcome of an evolution starting at $(i,r_1,r_2)$. That this function and the foliation is as smooth as the data follows from the existence of a spectral gap. The invariants of the evolution we found, reduce the calculation or estimation of this function to that of *s* at the free boundary points, and algebraic expressions in *i* and *r*.

What we have shown is that taking into account the heterogeneity of the infection process has an influence on the structure of epidemic models and thus on their outcome.

The example we presented here is a minimal example. The group size is minimal, there are only two infection rates, and there is only one homogeneous population. The discussion of this model can be seen as a proof of concept.

The same can be done for larger groups, with different types of individuals, and even infections by different variants of the virus. In such a case there is a normal form representation of the possible order of infection events. This then is a rooted tree, the root is the infectionless state, and the directed edges $e=(v,w)$ correspond to a new infection in the group. The vertex *v* corresponding to a state with *k*(*v*) infections, the vertex *w* to a state with $k(v) +1 = k(w)$ infections.

The general form of the *sir*-model will be as follows. The population consists of $j= 1, \ldots , M$ different types of groups with $n_j$ individuals. If those individuals are all distinguishable and there are *l* different variants of the virus, the tree will have $l \cdot ( n_j !)$ leaves, for all possible permutations.

In the $s-i$-formalism one has a semilinear equation of the form

$$\begin{aligned} \left( \partial _t \sum _{r=1}^{k(v)} \partial _{a_r} \right) i_v = R \quad \text{ in } \quad \mathbb {R}\times \Omega _{k(v)} \;, \; \Omega _{k(v)} = \{ 0 \le a_1 \le \cdots \le a_{k(v)} \} \end{aligned}$$

where *R* stands for the inhomogeneity in the semilinear equation, for each vertex *v*, see e.g. (Luckhaus and Stevens 2023). This will quickly become a case for computer algebra unfortunately. For indistinguishable individuals and one virus variant, instead of the tree, one obtains just a chain of length $n_j$ which is more manageable.

## Concluding remarks

Lockdown measures–as the data we discussed show–have not been able to halt the spread of Covid, not even if the asymptomatic individuals have a window of PCR positivity less than two days, as in the study on infection risk in public transport. And in such a case we can assume that $\alpha$ is positive for two days only, too. Here $\alpha$ is proportional to the virus load in the breath of an infectious individual.

One of the reasons for this failure of lockdown measures could be the heterogeneity of the infection scenarios, which we have analyzed here. Another reason is that the majority of infections is asymptomatic and therefore difficult to detect.

During the Wuhan lockdown, january to march 2020, nearly 4000 Covid deaths were officially counted (Weinland 2020). We can be sure, that only cases were counted, which were clearly attributable to Covid. And that would be one (the case of Covid pneumonia, i.e. the fully vaccinated victim) in three, if we take the example of the Pfizer study, compared to the way Covid deaths are counted currently. Moreover one has to take into account the partly chaotic situation in the Wuhan hospitals at the time. Multiplying the official death count by 4 in order to compare with the official Covid death count in Europe seems conservative. And using the IFR calculated for Geneva (Perez-Saez et al. 2020), one can estimate the number of infections from mid december to the end of february to have been 2 to 2.5 million people, i.e. ca. $15\%$ of the population of Wuhan.

If you enforce an even more stringent lockdown, with regular contacts restricted to small groups, that might work of course. But then the main problem will be to control the asymptomatic in group infection rate among the enforcers.

## Footnotes

1 Seroconversion means that a person develops specific antibodies.

## References

[CR1] Allerberger F (2021) Interview, June https://www.youtube.com/watch?v=f35yAcYnuGk

[CR2] Backhausz À, Balázs S (2022). Action convergence of operators and graphs. Can J Math.

[CR3] Becker NF, Dietz D (1995). The effect of household distribution on transmission and control of highly infectious diseases. Math Biosci.

[CR4] Bundesamt für Gesundheit (BAG) Schweiz https://www.bag.admin.ch

[CR5] Chandrashekar A (2020). SARS-CoV-2 infection protects against rechallenge in rhesus macaques. Science.

[CR6] Charité Research Organisaton (CRO) (2021) Studie zur Untersuchung des Corona-Infektionsrisikos im öffentlichen Personen-Nahverkehr. Ministry of Traffic of the german federal state of Baden Württemberg. https://vm.baden-wuerttemberg.de/fileadmin/redaktion/m-mvi/intern/Dateien/PDF/PM_Anhang/210510_%C3%96PNV_Studie_zum_Corona-Infektionsrisiko_im_%C3%96PNV.pdf

[CR7] Diekmann O, Heesterbeek H, Britton T (2012). Mathematical tools for understanding infectious disease dynamics.

[CR8] Feller W (1939). Die Grundlagen der Volterraschen Theorie des Kampfes ums Dasein in wahrscheinlichkeitstheoretischer Behandlung. Acta Bioth Ser A.

[CR9] Forien R, Pardoux E (2022). Household epidemic models and McKean–Vlasov Poisson driven stochastic differential equations. Ann Appl Prob.

[CR10] Hale J (1977). Theory of functional differential equations.

[CR11] Kermack WO, McKendrick AG (1936). The solution of sets of simultaneous integral equations related to the equation of Volterra. Proc London Math Soc (2).

[CR12] Knabel L et al (2021) High SARS-CoV-2 seroprevalence in children and adults in the Austrian ski resort of Ischgl. Nature Commun Med 1(4) 10.1038/s43856-021-00007-1 PMC8633917 34870284

[CR13] Luckhaus S (2020) Corona, Mathematical Epidemiology, Herd Immunity, and Data. MPI for Mathematics in the Sciences, Leipzig, Preprint 105

[CR14] Luckhaus S, Stevens A (2023) Kermack and McKendrick models on a two-scale network and connections to the Boltzmann equations. In: Mathematics Going Forward. Collected Mathematical Brushstrokes. Morel J-M, Teissier B(eds). Lecture Notes in Mathematics 2313, pp 399–408, Springer, Berlin

[CR15] McKendrick AG (1914). Studies on the theory of continuous probabilities, with special reference to its bearing on natural phenomena of a progressive nature. Proc London Math Soc.

[CR16] McKendrick AG (1926). Application of mathematics to medical problems.

[CR17] Jv Neumann (1928). Zur Theorie der Gesellschaftsspiele. Math Ann.

[CR18] Pardoux E (2016). Probabilistic models of population evolution.

[CR19] Perez-Saez et al (2020) Serology-informed estimates of SARS-CoV-2 infection fatality risk in Geneva, Switzerland. The Lancet 3099(20):30584–3 10.1016/S1473-3099(20)30584-3 PMC7833057 32679085

[CR20] Rass L, Radcliffe J (2003). Spatial deterministic epidemics.

[CR21] Rubin EJ, Longo DL (2020). SARS-CoV-2 Vaccination - An Ounce (Actually, Much Less) of Prevention. N Engl J Med.

[CR22] Stringhini et al (2020) Seroprevalence of anti-SARS-CoV-2 IgG anti- bodies in Geneva. The Lancet. 10.1016/S0140-6736(20)31304-0

[CR23] Thacker PD (2021) Covid-19: Researcher blows the whistle on data integrity issues in Pfizer’s vaccine trial BMJ 2021;375:n2635 10.1136/bmj.n2635 34728500

[CR24] Thieme HR (2003). Mathematics in population biology.

[CR25] Thomas SJ et al (2021) Safety and Efficacy of the BNT162b2 mRNA Covid-19 Vaccine through 6 Months. N Engl J Med 385:1761–1773 10.1056/NEJMoa2110345 PMC8461570 34525277

[CR26] Weinland D (2020) Inside Wuhan, Financial Times (25th of April)

[CR27] Westfälische Nachrichten (10th of Sept., 2021)

[CR28] Zou et al (2020) SARS-CoV-2 viral load in upper respiratory specimens of infected patients. NEJM 382:12. 10.1056/NEJMc2001737 10.1056/NEJMc2001737 PMC7121626 32074444

